# *In vivo* Trial of *Bifidobacterium longum* Revealed the Complex Network Correlations Between Gut Microbiota and Health Promotional Effects

**DOI:** 10.3389/fmicb.2022.886934

**Published:** 2022-06-17

**Authors:** You-Tae Kim, Chul-Hong Kim, Joon-Gi Kwon, Jae Hyoung Cho, Young-Sup Shin, Hyeun Bum Kim, Ju-Hoon Lee

**Affiliations:** ^1^Department of Food and Animal Biotechnology, Seoul National University, Seoul, South Korea; ^2^Department of Agricultural Biotechnology, Seoul National University, Seoul, South Korea; ^3^Center for Food and Bioconvergence, Seoul National University, Seoul, South Korea; ^4^Research Institute of Agriculture and Life Science, Seoul National University, Seoul, South Korea; ^5^Department of Food Science and Biotechnology, Graduate School of Biotechnology, Kyung Hee University, Yongin, South Korea; ^6^Food Research Center, Binggrae Co., Ltd., Namyangju, South Korea; ^7^Department of Animal Resources Science, Dankook University, Cheonan, South Korea

**Keywords:** *Bifidobacterium longum*, cholesterol reduction, anti-inflammation, gut microbiota, obesity

## Abstract

Complete genome sequence analysis of *Bifidobacterium longum* subsp. *longum* BCBL-583 isolated from a Korean female fecal sample showed no virulence factor or antibiotic resistance gene, suggesting human safety. In addition, this strain has oxygen and heat tolerance genes for food processing, and cholesterol reduction and mucin adhesion-related genes were also found. For *in vivo* evaluations, a high fat diet (HFD) mouse model was used, showing that BCBL-583 administration to the model (HFD-583) reduced the total cholesterol and LDL-cholesterol in the blood and decreased pro-inflammatory cytokines but increased anti-inflammatory cytokines, substantiating its cholesterol reduction and anti-inflammation activities. Subsequent microbiome analysis of the fecal samples from the HFD mouse model revealed that BCBL-583 administration changed the composition of gut microbiota. After 9 weeks feeding of bifidobacteria, Firmicutes, Actinobacteria, and Bacteroidetes increased, but Proteobacteria maintained in the HFD mouse models. Further comparative species-level compositional analysis revealed the inhibitions of cholesterol reduction-related *Eubacterium coprostanoligenes* and obesity-related *Lactococcus* by the supplementation of *B. longum* BCBL-583, suggesting its possible cholesterol reduction and anti-obesity activities. The correlation analysis of HFD-583 between the gut microbiota compositional change and cholesterol/immune response showed that Verrucomicrobia, Firmicutes, Actinobacteria, and Bacteroidetes may play an important role in cholesterol reduction and anti-inflammation. However, correlation analysis of Proteobacteria showed the reverse correlation in HFD-583. Interestingly, the correlation analysis of *B. longum* ATCC 15707 administration to HFD model showed similar patterns of cholesterol but different in immune response patterns. Therefore, this correlation analysis suggests that the microbial composition and inflammatory cytokine/total-cholesterol may be closely related in the administration of BCBL-583 in the HFD mice group. Consequently, BCBL-583 could be a good probiotic strain for gut health promotion through gut microbiota modulation.

## Introduction

The genus *Bifidobacterium* is one of the dominant colonizers in the human gastrointestinal tract (GIT). It is well-known that bifidobacteria may have various potential benefits for health promotion such as alleviation of diarrhea and constipation, immune regulation, anti-tumorigenesis, recovery of healthy gut microbiota after antibiotics treatment, and vitamin biosynthesis (Newbold, 1988; Perdigón et al., 2001; Honda et al., 2012; Jang et al., 2013; Bharti et al., 2017). These potential health benefits may be associated with several distinct characteristics of bifidobacteria: degradation of host-indigestible complex carbohydrates and plant-derived dietary fibers (Mayo, 1993; Gänzle and Follador, 2012), production of short-chain fatty acids (SCFAs) such as acetate and lactate *via* fructose-6-phosphate phosphoketolase (F6PPK) pathway for growth inhibition of acid-sensitive gut pathogens by lowering luminal pH (Petry et al., 2012; Ríos-Covián et al., 2016), colonization resistance on the mucus layer against infection of various pathogens in the human GIT (Bernet et al., 1993), and bacteriocin production (Lee et al., 2011; Liu et al., 2016). Therefore, bifidobacteria have been widely used as probiotics in functional food ingredients, dairy products, probiotics tablets, and even pharmaceutical additives.

In addition, immune regulation and cholesterol reduction have been suggested as the main health beneficial effects. As known previously, most of the immune responses in humans are associated with the human GIT system, where bifidobacteria is present as one of the major gut bacteria. It has been often reported that bifidobacteria may have a possible immune modulation activity with the whole cell, cell membrane, cell extract, or even CpG motif in their DNA. A human feeding study on whole cell supplementation of *Bifidobacterium longum* BB536 to elderly subjects showed that the serum IgA was increased, and the natural killer cell activity was maintained, probably due to its immune stimulation (Akatsu et al., 2013). In addition, whole cell supplementation of *B. bifidum* LMG13195 to systemic lupus erythematosus (SLE) patients prevented CD4^+^ lymphocyte over-activation and reduced Interleukin (IL)-6 and Interferon (IFN)-γ in the serum suggesting an anti-inflammation activity (López et al., 2016). Furthermore, the CpG-rich sequences of *B. longum* NCC2705 induced the production of monocyte chemoattractant protein 1 (MCP-1) and tumor necrosis factor (TNF)-α by stimulation of Toll-like receptor 9 (TLR9) in RAW 264.7 macrophages (Ménard et al., 2010).

Cholesterol as a type of lipid is an important precursor of several steroid hormones (progesterone, estrogen, and testosterone), bile acid, and vitamin D (Makishima, 2005). However, a high concentration of cholesterol may increase the risk of heart attack, stroke, and even blood clot formation in the blood stream by hypercholesterolemia (Egan et al., 2013). Interestingly, bifidobacteria have been reported to have an anti-cholesterolemic effect. It is suggested that bifidobacteria reduce blood cholesterol using two different ways: (a) bifidobacterial cells absorb and accumulate intestinal cholesterol, and they are gradually excreted with the feces from the intestine (Dambekodi and Gilliland, 1998; Tok and Aslim, 2010); (b) cholesterol is a precursor of conjugated bile acid. Bifidobacteria have bile acid hydrolase to convert conjugated bile acid to an unconjugated one. After deconjugation of bile acid, this unconjugated one is precipitated and excreted with the feces from the intestine. To recover the level of bile acid, cholesterol is gradually converted to bile acid in the liver, resulting in cholesterol reduction in the blood (Walker and Gilliland, 1993; Tsai et al., 2014). Although the exact mechanisms of cholesterol reduction by bifidobacteria are not clearly understood yet, these cholesterol reduction studies have suggested other potential cholesterol reduction mechanisms: incorporation of cholesterol into the cellular membrane (Dambekodi and Gilliland, 1998) and alteration of the cholesterol structure in the bacterial cell (Miremadi et al., 2014).

Experimental determination of bile salt deconjugation and cholesterol assimilation activities with four different *B. longum* strains (CCFM 1077, I3, J3, and B3) from the elderly showed strain specificity *in vitro*, suggesting that *B. longum* has two different cholesterol reduction mechanisms, bile salt deconjugation by bile salt hydrolase and/or cholesterol assimilation by specific absorption as mentioned previously. To evaluate them, each *B. longum* strain was administrated to a high-cholesterol diet rat model which revealed *in vivo* cholesterol reduction from 27% to 44% supporting these mechanisms (Jiang et al., 2021). Interestingly, the administration of *B. longum* CCFM1077 to the rat model showed the highest cholesterol reduction activity because it has both bile salt hydrolase and cholesterol assimilation activities. In addition, an *in vitro* cholesterol reduction test with *B. bifidum* PRL2010 and its *in vivo* administration to a mouse model showed this cholesterol reduction activity (Zanotti et al., 2015). Further transcriptional analysis revealed that genes encoding putative transporters and reductases were up-regulated, suggesting cholesterol assimilation. In addition, *B. pseudocatenulatum* CECT 7765 with a cholesterol reduction activity was administrated to high fat diet (HFD) fed mice to evaluate the effect of the immune response (Cano et al., 2013). Interestingly, IL-6 and MCP-1 were significantly decreased but IL-4 was increased, suggesting a possible anti-inflammation activity. Moreover, 14% of the total serum cholesterol in CECT 7765-fed HFD group was reduced compared to that of only the HFD group. This result suggests a possible correlation between the anti-inflammation activity and cholesterol reduction activity by *B. pseudocatenulatum* CECT 7765. Therefore, bifidobacteria may have a cholesterol reduction potential for immune modulation. However, this correlation of cholesterol reduction and immune response is not clearly understood yet. Therefore, this interaction between them by bifidobacteria needs to be elucidated on the omics level.

To clarify this, a new probiotic strain, *B. longum* BCBL-583 was initially isolated from a Korean female fecal sample and its cholesterol reduction activity and immune response were evaluated *in vitro* (Yi et al., 2018). To further understand its correlation of cholesterol reduction and immune response on the omics level, genomic characterization and transcriptomic analysis were carried out to identify the mechanism of cholesterol reduction by *B. longum* BCBL-583. Furthermore, an *in vivo* feeding study and microbiome analysis with a mouse model by administration of *B. longum* BCBL-583 were performed to evaluate the relationship of the serum cholesterol reduction and intestinal bacteria composition. This study provides useful scientific information on the correlation between cholesterol reduction and immune regulation by the administration of bifidobacteria. Thus, this strain could be a good candidate as a new functional food additive for serum cholesterol reduction and anti-inflammation in humans.

## Materials and Methods

### Bacterial Strains and Growth Condition

*Bifidobacterium longum* subsp. *longum* BCBL-583 was previously isolated from a 25-year-old healthy Korean female and stored in bifidobacteria culture collection of Food Microbiome Laboratory, Seoul National University (Seoul, South Korea). And then, this strain was selected from 753 *Bifidobacterium* strains in bifidobacteria culture collection and tested for the development of a new probiotic strain (Yi et al., 2018). *Bifidobacterium longum* subsp. *longum* ATCC 15707 was selected for comparative functional analysis as a control strain. All *Bifidobacterium* strains were cultivated in de Man-Rogosa-Sharpe medium supplemented with 0.05% (wt/vol) L-cysteine hydrochloride (MRSc) at 37°C for 24 h under anaerobic condition. Culture media and all chemical reagents were purchased from Difco (United States) and Sigma-Aldrich (United States).

### Genome Sequencing and Bioinformatic Analysis

BCBL-583 cells were harvested by centrifugation at 10,000 × *g* for 5 min after cultivation. The genomic DNA was extracted and purified using the G-Spin Total DNA Extraction Kit (iNtRON Biotechnology, Korea), according to the manufacturer’s protocol. The genome of BCBL-583 was completely sequenced using PacBio RS II (Pacific Biosciences, USA) by Macrogen, Inc. (South Korea). The qualified raw reads were assembled using canu-2.0 with the default parameters (Larsen et al., 2014; Koren et al., 2017). The open reading frames (ORFs) in the complete genome sequence were predicted using a combination of GeneMarkS2 (Lomsadze et al., 2018) and Glimmer3 (Delcher et al., 2007). The positions of the ribosome binding sites (RBSs) were predicted using RBSfinder (Suzek et al., 2001). Functions of the ORFs were predicted using the BLASTP-GAMOLA and InterProScan5 programs (Altermann and Klaenhammer, 2003; Jones et al., 2014). The tRNA and rRNA genes were predicted by the ARAGORN and RNAmmer programs (Laslett and Canback, 2004; Lagesen et al., 2007). The functional categorizations and metabolic pathways were determined by COGNIZER and KASS, respectively (Moriya et al., 2007; Bose et al., 2015). Toxin and virulence factors were detected using the virulence factor database (VFDB; Liu et al., 2019) and antibiotic resistance genes with the comprehensive antibiotic resistant database (CARD; Alcock et al., 2020). All programs used for the bioinformatic analysis were run on a Linux system.

### Transcription Analysis of the Bile Salt Hydrolase Gene

To compare mRNA expression profiles regarding bile salt hydrolase, BCBL-583 was cultivated in the MRSc medium with 0.3%, 0.15%, and 0.075% of bile acid (Sigma-Aldrich). After cultivation for 18 h at 37°C, the cells were collected and treated with RNA*later* (Thermo Fisher Scientific). Total RNA for each condition was isolated using the RiboPure RNA Purification Kit (Thermo Fisher Scientific) following the manufacture’s protocol. The mRNA expression levels were analyzed with the One Step TB Green® PrimeScript™ RT-PCR Kit II (TaKaRa, Japan) on the CFX96 system (Bio-Rad, United States). The sequences of the primers are listed in Supplementary Table S1. Quantitative PCR was performed with the following cycle: initial reverse transcription at 42°C for 5 min before 95°C for 10 s and then 40 cycles at 95°C for 5 s and 60°C for 30 s. The cycle threshold (*C_T_*) was determined automatically using the CFX Manager™ Softerware version 3.1 (Bio-Rad). All samples were analyzed in triplicate. Expression levels were normalized, and the Real Time (RT)-PCR data were analyzed by the 2^−ΔΔ*CT*^ method (Livak and Schmittgen, 2001).

### Measurement of Cholesterol Accumulation *in vitro*

*Bifidobacterium longum* BCBL-583 was cultivated in MRSc medium with or without 0.03% Cholesterol-Water Soluble powder (Sigma-Aldrich) to determine the cholesterol content in the medium and membrane/cytoplasm of the bacterial cell after incubation at 37°C for 18 h. The cell was harvested by centrifugation at 10,000 × *g* for 2 min, and the cholesterol content of supernatant was estimated. The harvested cells were resuspended with 2 ml of distilled water followed by sonication using EpiShear Probe Sonicator (10 s/10 s pulse for 5 min, 28% amplitude; Active Motif, United States) in an ice bath to obtain cytoplasm. The cellular membrane was isolated according to the method described by Thorne and Barker (1972). The cholesterol quantification assay was carried out using a colorimetric method as previously described with minor modifications (Miremadi et al., 2014). Briefly, 1 ml of the supernatant of the medium, cell extract, or membrane extract was mixed with 1 ml of KOH (33%, w/v) and 2 ml of 96% ethanol. The mixture was vortexed thoroughly and then incubated at 37°C for 15 min. After cooling to room temperature, 2 ml of molecular water and 3 ml of hexane (Sigma-Aldrich) were added to the mixture followed by vortexing for 1 min. The hexane layer was then separated from the mixture and transferred to a fresh tube for evaporation in the Savant SpeedVac SPD1030 (Thermo Fisher Scientific, United States). The residues were dissolved by o-phthalaldehyde reagent (50 mg OPA dissolved in 100 ml glacial acetic acid). And then, 0.5 ml of 98% sulphuric acid (Sigma-Aldrich) was added and vortexed for 1 min. After vortexing, color development of cholesterol for 10 min was performed at room temperature. The cholesterol concentration was read by measuring the absorbance at 550 nm using a DU730 spectrophotometer (Beckman Coulter, United States).

### Animal Models and Strain Feeding

**Figure 1:**
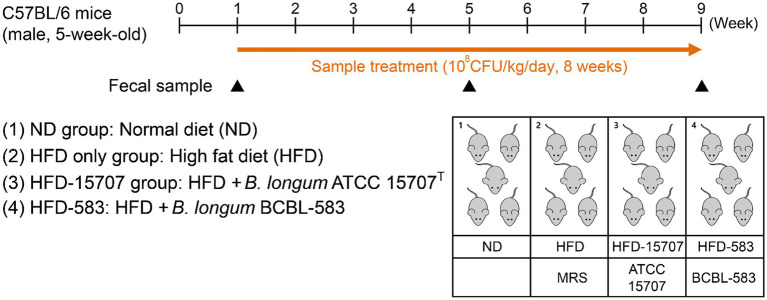
Experimental procedure for high fat diet (HFD) model and administration of bifidobacteria. Mice were given a single week for normal diet (ND) and then randomly divided into four groups. Each bacterial sample was administered orally in the mice (10^8^ CFU/kg/day).

Male C57BL/6 mice (5-week-old) were obtained from RaonBio (South Korea) and acclimatized to laboratory conditions consisting of a 12:12-h light–dark cycle, 24°C, and 55% humidity for 1 week with a normal diet of D12450B (10 kcal% fat, Research Diet, United States; Supplementary Table S2) as a pre-treatment period. At the first week (Week 1) of treatment, five mice were randomly picked and grouped into one of the following five groups: ND group [normal diet (ND) without MRSc medium], HFD only group [HFD with D12492 (HFD; 60 kcal% fat, Research Diet; Supplementary Table S2) and MRSc medium], HFD-15707 group (HFD with MRSc medium and *B. longum* ATCC 15707), and HFD-583 group (HFD with MRSc medium and *B. longum* BCBL-583). The added amount of specific strain was 10^8^ CFU/kg/day in the group, and total feeding period was 8 weeks (Figure 1). All samples were administered *via* oral gavage using a disposable oral zonde (RaonBio) attached to 1 ml syringe. For the gavage, two *Bifidobacterium* strains were cultivated until an optical density of 1.0 at 600 nm wavelength and then administered to the mice in each group every day. The mice body weight was measured twice a week until the end of this study.

### Cholesterol Levels in the Serum

After 8 weeks of the experimental diets, the mice were subjected to fasting for 12 h and then sacrificed. Blood samples were collected from the heart, centrifuged at 3,000 × *g* for 15 min and stored at −80°C. The measurements of the serum total cholesterol and HDL-/LDL-cholesterol levels were performed using a Total Cholesterol Assay kit and HDL and LDL/VLDL Cholesterol Assay kit (Cell Biolabs, United States), according to the manufacturer’s instructions.

### Cytokine Assay

To determine the cytokine levels in the spleen, it was excised after the mice was euthanized. The excised spleen was soaked in 3 ml of ice-cold Hank’s Balanced Salt Solution (HBSS) buffer (Thermo Fisher Scientific) for 1 h. It was poured onto a clean plate and chopped with a sterilized scalpel. Chopped tissue in HBSS buffer was passed through 70 mash cell strainer (SPL Life Science, Korea), and then, 10 ml of ammonium chloride solution (STEMCELL Technologies, Canada) was added to the pass through. After incubation at 4°C for 10 min to lyse the red blood cells, a pellet was obtained by centrifugation in 300 × *g* for 5 min at 4°C. The pellet was resuspended to 1 ml of RPMI-1640 (supplemented with 10% FBS and 2 mM glutamine, WELGENE, South Korea). After cell counting, the resuspended spleen cells were inoculated into fresh RPMI-1640 medium and incubated at 37°C for 72 h with aeration supplemented with 5% CO_2_. The cultivated cells were harvested using a sterilized cell scraper, and the supernatant was separated by centrifugation at 3,000 × *g*. The supernatant was used for estimating the cytokine levels of tumor necrosis factor (TNF)-α, interleukin (IL)-6, IL-10, and IL-4 by ELISA kit (Koma Biotech, South Korea).

### Microbiome Analysis

Stool samples from each mouse were collected at the first days of Week 1, Week 5, and Week 9 and stored at −80°C. Before the extraction of the stool DNA using QIAamp DNA Stool Mini Kit (Qiagen, United States), the stool samples were soaked in 1.4 ml of ASL lysis buffer with stainless beads (GeneReach Biotechnology, Taiwan) and boiled for 10 min. After boiling, the mixtures were vortexed vigorously and centrifuged for 1 min at 10,000 × *g*, respectively. The supernatant was obtained, and the manufacturer’s instructions were followed to extract the stool DNA. All extracted total fecal DNA was quantified with the NanoDrop 2000 (Thermo Fisher Scientific) and diluted to 5 ng/μl to amplify the 16S rRNA gene by PCR.

The diluted total fecal DNA was used for PCR amplification with universal primers targeting from V3 to V4 regions of the 16S rRNA gene. For amplification of the 16S rRNA, the 341_F (CCTACGGGNGGCWGCAG) and 805_R (GACTACHVGGGTATCTAATCC) universal primer set with sample-specific barcodes was used as in a previous study (Herlemann et al., 2011). The amplification was performed using KAPA HiFi HotStart ReadyMix (Roche, United States), and 16S rRNA amplicons were extracted by the AxyPrep DNA Gel Extraction Kit (Axygen Bioscience, United States). Their sequences were analyzed using the Illumina MiSeq system with 2 × 301 bp paired-end sequencing method following the manufacturer’s instructions.

The qualified sequence reads were processed with the following steps: (i) the paired end fastq sequence set of each sample was aligned by the “fastq-join” method using the default parameters (Aronesty, 2013); (ii) multiple joined FASTQ sequences were demultiplexed by “multiple_split_libraries_fastq.py” script in the QIIME pipeline program; (iii) chimeric sequences of each split library were identified and filtered using UCHIME version 4.2.40 (Edgar et al., 2011) with 97% identity in the SILVA v132 database using the default parameters, and (iv) picking of the open reference operational taxonomy units (OTUs) was performed by the “pick_otu_reference_otus.py” script in the QIIME pipeline program with the 16S database provided by SILVA as a reference to the OTUs. All samples were normalized to the smallest read number sample by randomly chosen reads. To calculate the α-diversity of the total samples, Shannon, Simpson, and Richness indexes were analyzed in the QIIME script, and core diversity analysis was performed for the β-diversity using the default parameters. Additional analyses and visualization were performed using R studio (RStudio, United States) and related packages.

### Statistical Analysis

All data are expressed as the mean ± SD. Statistical significance was assessed by Duncan’s multiple range test and Student’s *t*-test. IBM SPSS software version 25 (United States) was used to perform all statistical tests. A value of *p* < 0.05 was considered statistically significant.

## Results

### Genomic Characterization of *Bifidobacterium longum* BCBL-583

#### General Genome Information

**Figure 2:**
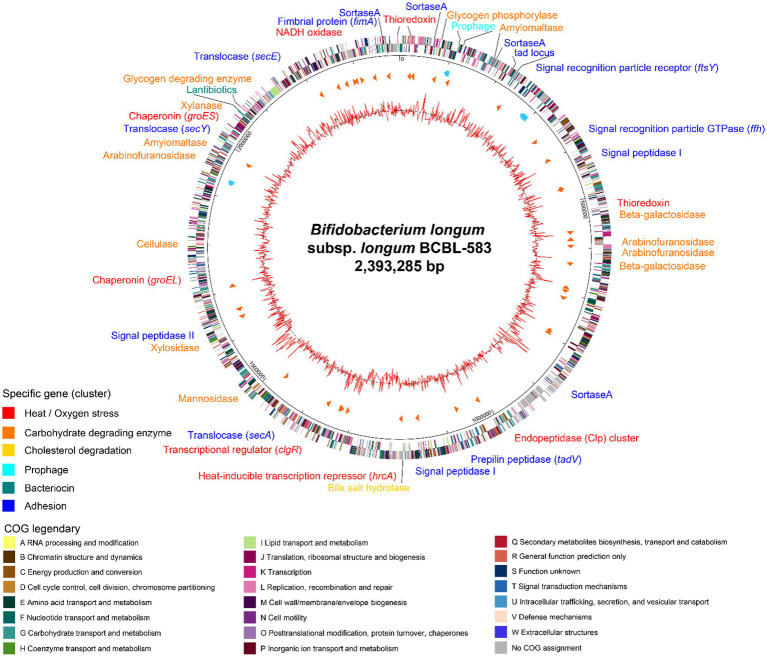
Circular genome map of *Bifidobacterium longum* BCBL-583. The outer circle indicates the location of all annotated open reading frames (ORFs) in double strands colored by clusters of orthologous groups (COG), and the inner circle with red peaks indicates GC content. Between these circles, sky blue arrows indicate the rRNA operons, and orange arrows indicate the tRNAs. Specific genes or gene clusters were colored according to their functions in upper legend. The colors of COG categories were indicated in lower legend.

The complete genome sequence of *B. longum* BCBL-583 has a circular chromosomal DNA of 2,393,285 bp with no plasmid (Figure 2). The GC content of the chromosome is 60.8% like other bifidobacterial genomes with a high GC content (Lee and O’Sullivan, 2010). In total, 1,942 ORFs were predicted with 56 tRNAs and 12 rRNAs (4 rRNA operons). Among the total predicted ORFs, functions of 1,293 ORFs (66.6%) were predicted. In addition, a probable prophage was predicted between *attL* and *attR* sequences (BCBL_0084 to BCBL_0095) including phage tyrosine-type recombinase and integrase but no reverse transcriptase and phage structural proteins, suggesting that it may be fragmented. In addition, VFDB and CARD analyses of this genome showed that no genes encoding toxins, virulence factors, or complete antibiotic resistance genes were detected in this genome, suggesting the safety of BCBL-583 for human applications.

#### Carbohydrate Utilization

The genome annotation revealed that this strain might have specific functions such as complex carbohydrate degradation, cell adhesion, bacteriocin production, and cholesterol reduction (Supplementary Table S3). This genome encodes specific genes associated with 26 host-indigestible complex carbohydrates metabolic enzymes for degradation of oligosaccharides and plant-derived dietary fibers including xylan, arabinofuran, and arabinogalactan, suggesting that this intestinal strain BCBL-583 has a good adaptation capability in the human gut habitat (Lee and O’Sullivan, 2010).

#### Cell Adhesion and Secretion Systems

To colonize on the mucus layer in the gut environment for survival, cell adhesion activity may be important for bifidobacteria. This genome has cell adhesion-associated protein secretion systems and encodes extracellular proteins related to mucus layer anchoring. Therefore, extracellular proteins containing a signal peptide sequence and the LPXTG motif are secreted from the cell membrane by a specific extracellular protein secretion system [signal recognition particle (SRP)-dependent proteins and Sec-dependent secretion system] and anchoring-process and maturation enzymes (Type I and Type II signal peptidase and sortase A; Dalbey and Chen, 2004). Once an extracellular protein is produced in the cells, SRP binds to a signal peptide of the extracellular protein and leads it to the SRP receptor (BCBL_0185) to export the protein from the cell membrane using Sec translocase (BCBL_1133, 1,213, 1717, and 1824). Then, the signal peptide region of the secreted protein is cleaved and removed by signal peptidase (BCBL_0356, 1,033, and 1,353), and sortase A (BCBL_0049, 0132, 0750, and 1976) recognizes and activates a C-terminal LPXTG motif in the secreted protein for anchoring to peptidoglycan (Ton-That et al., 1999). In addition to this cell-adhesion mechanism of bifidobacteria, a tight adherence (*tad*) locus (BCBL_0122 to BCBL_0128) was detected in the genome, probably involved in the construction and localization of a pilus (Motherway et al., 2011). Among the *tad* locus, *tadA* gene encoding the Flp pilus assembly ATPase component (BCBL_0123) has a role for the secretion of putative pilin proteins in the *tad* locus (BCBL_0126 and 0127) or FimA prepilin (BCBL_1977) using sortase A (BCBL_1976).

#### Lantibiotic Gene Cluster

In this genome, interestingly, a 10.2 kb gene cluster was detected which carries eight genes for lantibiotic production. This gene cluster consists of genes encoding a two-component signal transduction system (BCBL_1800, transcription regulator; BCBL_1799, histidine kinase), a lantibiotic prepeptide (BCBL_1798), a lantibiotic response regulator (BCBL_1797), lantibiotic modification enzymes (BCBL_1796 and BCBL_1795), a lantibiotic immunity protein (BCBL_1794), and a transporter with a protease domain for the secretion of the peptide (BCBL_1793; Klaenhammer, 1993). The structure of this cluster is the same as that of *B. longum* subsp. *longum* DJO10A with an average protein homology of 98.5% (Lee et al., 2011).

#### Oxidative and Temperature Stresses

Bifidobacteria encounter oxidative and temperature stress when utilized for food industrial processes. They are obligate anaerobic bacteria and have transient oxidative stress resistance using NADH oxidase (BCBL_1944) and thioredoxin systems (BCBL_0026, 0418, 2004) to convert reactive oxygen species (ROS) to H_2_O (Arnér and Holmgren, 2000). In addition, bifidobacteria have heat-shock response regulators and heat-shock proteins for the prevention of protein denaturation by heat. This genome has the HrcA-CIRCE (controlling inverted repeat of chaperon expression; BCBL_1124) system with the consensus repeat sequence for heat-shock proteins (*groES*, BCBL_1771; *groEL*, BCBL_1482; Baldini et al., 1998) and the Clp transcriptional regulator (*clgR*, BCBL_1219) for the Clp gene cluster (BCBL_0843 to 0845; Ventura et al., 2005), respectively.

#### Cholesterol Reduction

It has been known that bifidobacteria may lower the cholesterol concentration (Walker and Gilliland, 1993). Interestingly, this genome has a gene encoding bile salt hydrolase (*bsh*; BCBL_1011), probably associated with deconjugation and precipitation of bile salt. Due to the removal of bile salt, intestinal cholesterol should be converted to bile salt to maintain a constant level, resulting in cholesterol reduction. To further understand cholesterol reduction by BCBL-583, the systematic response to cholesterol needs was studied *in vitro*.

### Predicted Cholesterol Reduction Mechanism of *Bifidobacterium longum* BCBL-583

**Figure 3:**
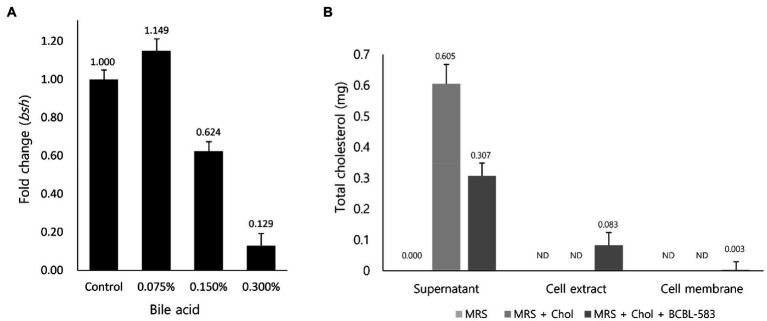
Transcription analysis of *bsh* gene to bile acid and cholesterol adsorption assay of BCBL-583. **(A)** The *bsh* gene expression levels were quantified in the different concentration of bile acid by qRT-PCR. **(B)** To clarify the cholesterol adsorption ability of BCBL-583, total cholesterol amounts were determined in supernatant, cell extract, and cell membrane.

As previously mentioned, two hypotheses regarding cholesterol reduction by bifidobacteria were suggested: (1) compensation for a lowered concentration of bile acid after deconjugation in blood by bioconversion of cholesterol and (2) absorption and accumulation of intestinal cholesterol in the bifidobacterial cells. To clarify the first hypothesis, transcription profiles of the *bsh* gene in BCBL-583 were determined using qRT-PCR with different concentrations of bile acid. A low concentration of bile acid (0.075%) increased the *bsh* gene transcription, but a high concentration (0.15% and 0.3%) decreased the RNA expression, suggesting that mRNA expression of the *bsh* gene may not be bile acid concentration-dependent, even though a low concentration of bile acid stimulated the mRNA expression (Figure 3A). Based on this result, the first hypothesis may be not a major cholesterol reduction mechanism in the strain BCBL-583. In addition, a subsequent signal peptide analysis of the *bsh* gene revealed that this gene does not have a signal peptide, indicating that this gene is not an extracellular protein (Data not shown) like in previous reports (Dong et al., 2013). Therefore, the mRNA expression of the *bsh* gene under a lower concentration of bile acid suggests that BCBL-583 may have specific transporters associated with bile acid transport into the cells. However, the specific bile acid transport was not clearly detected in the complete genome sequence, probably due to insufficient annotation data of the *B. longum* genomes. Subsequently, the second hypothesis was also evaluated to determine the cholesterol absorption and accumulation activities of BCBL-583. After incubation of BCBL-583 with 0.5% cholesterol, the quantification assay of cholesterol in the culture media showed a 50.7% reduction after incubation. To evaluate the absorption and accumulation of cholesterol, BCBL-583 cells were disrupted and separated into cell extract and cell membrane. The subsequent cholesterol assay revealed that most of the cholesterol was detected in the cell extract, suggesting that cholesterol may be absorbed and may accumulate inside of the cells (Figure 3B). However, a cholesterol-specific transporter was not detected in the complete genome because of the same reason for a bile acid transporter. Based on these two results, the cholesterol reduction of the strain BCBL-583 may be accomplished by the second hypothesis. These results confirmed that BCBL-583 has a cholesterol reduction activity using *in vitro* adsorption and accumulation of cholesterol in the cells. Further *in vivo* experiments should be conducted to substantiate this suggestion.

### Cholesterol Reduction in the Serum of an HFD Mouse Model

**Figure 4:**
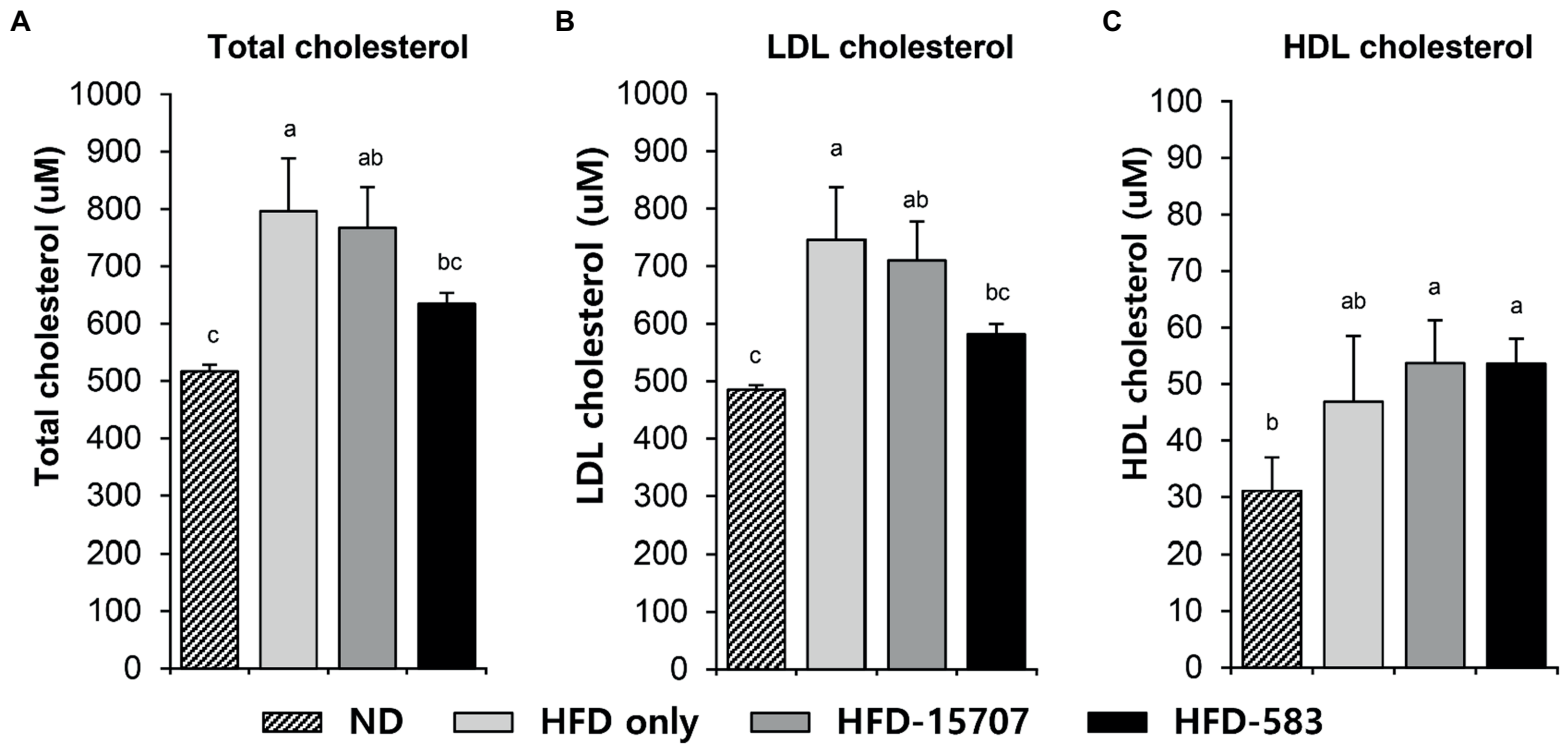
Cholesterol levels in serum samples of four groups after 9 weeks. **(A)** Total cholesterol level, **(B)** LDL cholesterol level, and **(C)** HDL cholesterol level in serum. Differential letters indicate statistically relevant differences among the different groups (*p* < 0.05).

While bile acid is generally recycled between the liver and intestine, its deconjugation by bile salt hydrolase of intestinal bacteria causes the loss of bile acid. To compensate for this loss of bile acid, cholesterol is converted to bile acid in the liver, resulting in cholesterol reduction in the liver. This can affect and lower the levels of LDL-cholesterol in blood because cholesterol is also recycled between the liver and tissues by the bloodstream. However, HDL-cholesterol recycles from the tissues or blood to the liver to maintain the level of cholesterol by conversion in the liver. To validate the cholesterol reduction by *B. longum* BCBL-583, the levels of total cholesterol, LDL-cholesterol, and HDL-cholesterol in the blood samples of the HFD-583 mouse group were determined. The normal diet (ND) and HFD groups had a cholesterol level of 0.0014% and 0.0228% (Supplementary Table S2), respectively, indicating that the HFD group had 16.7-fold more cholesterol than the ND group. Consistent with the cholesterol amounts in the diets, total cholesterol assay between these two groups showed that the mouse blood samples of the ND group had the lowest total cholesterol level compared to those of the other HFD groups (Figure 4A). To clarify the cholesterol reduction activities of *B. longum*, the total cholesterol assay was performed with the mouse blood samples of the HFD only, HFD-15707, and HFD-583 groups for comparison. Compared to the HFD only group, 3.62% and 20.33% of cholesterols were reduced in the blood samples of the HFD-15707 and HFD-583 groups, respectively, indicating that *B. longum* BCBL-583 has the highest cholesterol reduction activity (Figure 4A). Furthermore, a subsequent LDL-cholesterol assay showed that 4.82% and 22.06% of LDL-cholesterols were reduced in the blood samples of the HFD-15707 and HFD-583 groups, respectively, probably due to the cholesterol reduction (Figure 4B). However, the levels of HDL-cholesterol were not reduced by *B. longum* because HDL-cholesterol in the blood returns to the liver to compensate for the reduced level of cholesterol (Figure 4C).

### Immune Regulation of *Bifidobacterium longum* BCBL_583 in the Mouse Spleen

By the administration of the HFD, white adipose tissue is activated to store triglyceride and to secrete adipokines and cytokines (Toren et al., 2013). Moreover, HFD-derived free fatty acids impair the immune system further increasing the risk of inflammation (Tanaka et al., 2020). From this mechanism, macrophages and dendritic cells in the spleen secrete inflammatory cytokines, which stimulate the production of inflammatory cells in the bone marrow (Tall and Yvan-Charvet, 2015).

**Figure 5:**
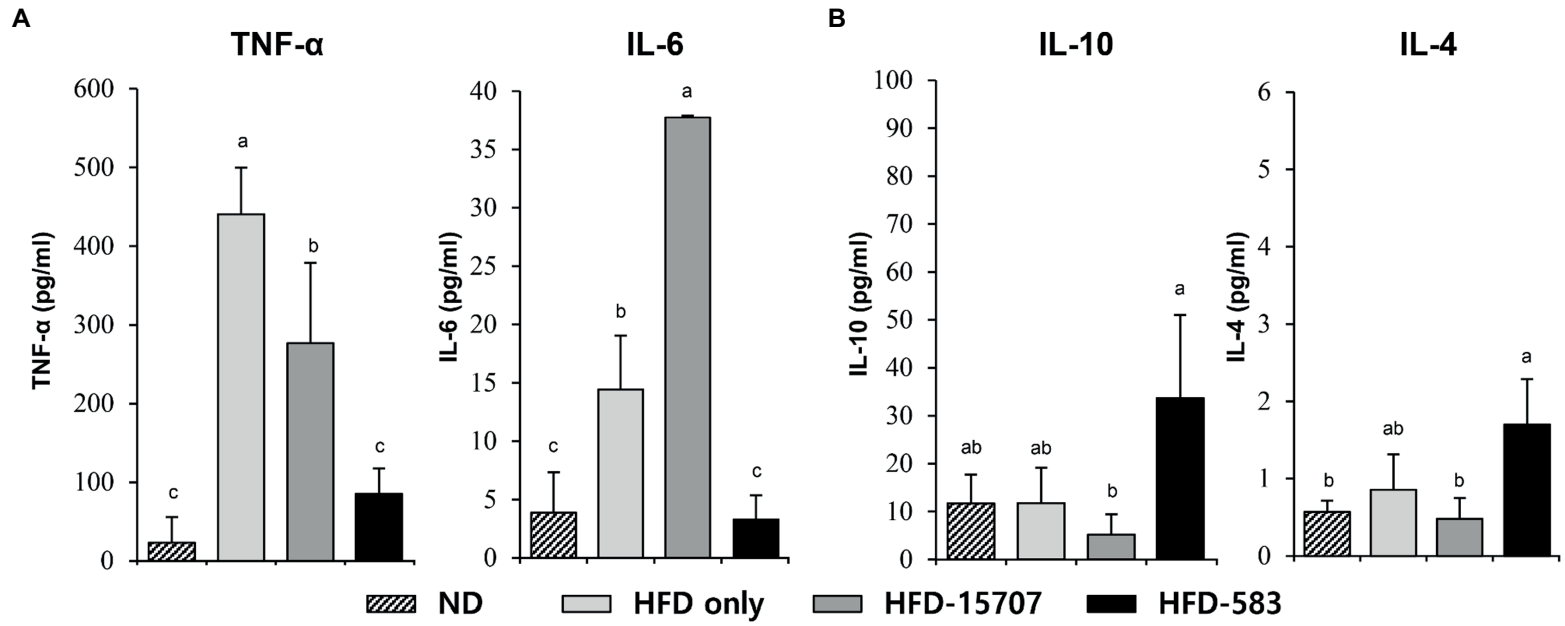
Inflammatory cytokine levels from spleen. **(A)** Pro-inflammatory cytokines: tumor necrosis factor (TNF)-α and interleukin (IL)-6, **(B)** anti-inflammatory cytokines: IL-10 and IL-4 levels from spleen. Differential letters indicate statistically relevant differences among the different groups (*p* < 0.05).

Based on this, the production of inflammatory cytokines was determined from the spleens of the ND and HFD groups. Compared to the level of pro-inflammatory cytokines in the ND group, the levels of TNF-α and IL-6 in the HFD only group were increased 19.1-fold and 3.7-fold, respectively (Figure 5A). However, TNF-α (37.2%) was decreased, and IL-6 (261.5%) was increased in the HFD-15707 group compared to the HFD only group. In addition, the TNF-α and IL-6 were reduced to 80.6% and 77.2% in the HFD-583 group, respectively (Figure 5A). However, the production patterns of the anti-inflammatory cytokines (IL-10 and IL-4) were different in the HFD groups. The HFD only and HFD-15707 groups did not show any increment of these anti-inflammatory cytokines, but they were reduced in the HFD-15707 group (56.1% and 43.8%, respectively; Figure 5B). Interestingly, the levels of the anti-inflammatory cytokines were increased in the HFD-583 group (286.7% and 198.7%, respectively). Based on this result, BCBL-583 administration to the HFD mouse model may suppress inflammation compared to the type strain of *B. longum* (ATCC 15707; Medina et al., 2007).

### Compositional Change of the Gut Microbiome With BCBL_583

#### Impact of *Bifidobacterium longum* Supplementation for Compositional Change of Gut Microbiota

**Figure 6:**
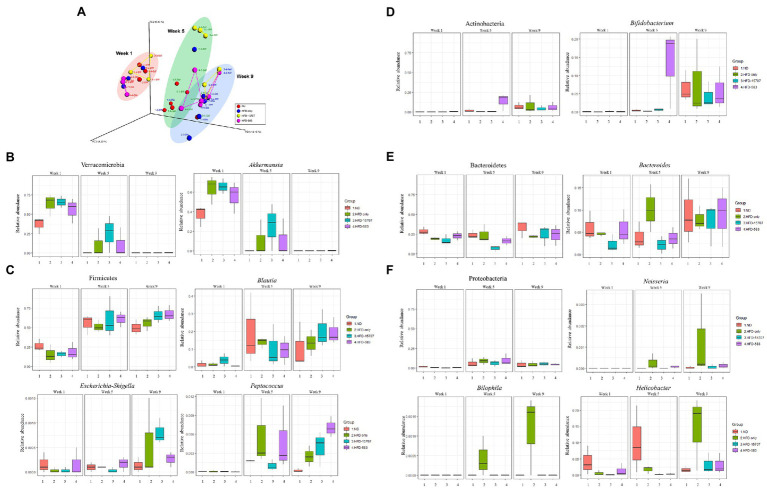
Principal coordinates analysis (PCoA) and microbial compositional changes of representative phyla and genera of four groups in Week 1, Week 5, and Week 9. **(A)** β-Diversity plot using UniFrac distance with four groups. **(B–F)** The major phyla and genera were indicated on the box plot.

For the microbiome analysis of the mouse gut, three mice were randomly selected in each group and their fecal samples were collected at the first days of Week 1, Week 5, and Week 9. After Illumina MiSeq sequencing of extracted DNA from the fecal samples, qualified sequence reads were obtained and normalized (Supplementary Figure S1). To understand the impact of *B. longum* BCBL-583 on the gut microbiota, compositional changes of the test mice gut microbiota were compared using Principle Coordinate Analysis (PCoA) based on the unweighted UniFrac distance (Figure 6A). This plot showed that clusters were moved from left to right according to the weeks passed in all groups. However, the passages of the ND and HFD groups were slightly different, suggesting that HFD might be important for the change of the microbiome in the test mice. Among the HFD groups, the passages of the bacterial feed groups (HFD-15707 and HFD-583) were also different from that of the HFD only group, suggesting that administering *B. longum* might have an impact on the change of gut microbiota in test mice. To elucidate the impact of the *B. longum* administration, the compositional changes of the gut microbiota in the test mice were compared on the phylum and genus level. Five major phyla and their associated major genera were selected and compared: Verrucomicrobia (*Akkermansia*; Figure 6B), Firmicutes (*Blautia*, *Escherichia-Shigella*, *Peptococcus*; Figure 6C), Actinobacteria (*Bifidobacterium*; Figure 6D), Bacteroidetes (*Bacteroides*; Figure 6E), and Proteobacteria (*Neisseria*, *Bilophila*, *Helicobacter*; Figure 6F). The comparative compositional changes in the five phyla showed that the compositions of Firmicutes, Actinobacteria, Bacteroidetes, and Proteobacteria increased from Week 1 to Week 9 but that of Verrucomicrobia decreased during this period. Interestingly, this trend was also observed even in their associated genera. A previous study regarding obesity and gut microbiota revealed that obesity may be associated with the compositional reduction of *Akkermansia* supported by the significant compositional reductions of Verrucomicrobia and its *Akkermansia* in all HFD groups (Figure 6B; Everard et al., 2013). Notably, the overall compositions of the phylum Proteobacteria and its associated genera increased. However, for the compositions of the HFD groups, all associated genera (*Neisseria*, *Bilophila*, and *Helicobacter*) were significantly reduced in the *B. longum*-fed groups (HFD-15705 and HFD-583), suggesting that *B. longum* may have an inhibition activity against these genera bacteria (Figure 6F). It was previously reported that the growth of *Helicobacter* was inhibited by an HFD supplemented with *Bifidobacterium*, supporting this result (Wang et al., 2004). Based on these results, HFD reduced the composition of Verrucomicrobia but increased the compositions of the other four major phyla. In particular, administering *B. longum* showed a significant impact on the growth inhibition of the associated bacteria of the phylum Proteobacteria.

#### Effect of Gut Bacteria Modulation for Obesity and Cholesterol Reduction

To further understand the compositional change of gut bacteria regarding obesity and cholesterol reduction, compositional change of gut microbiota was re-analyzed in the species level. The species of only 22 genera from compositional analysis data of gut microbiota were identified (Supplementary Table S4). Among them, compositional changes of species in the major genera of *Bifidobacterium*, *Eubacterium*, *Lactococcus*, and *Streptococcus* were monitored and compared. Previously, it was reported that *Eubacterium coprostanoligenes* is a cholesterol-reducing bacterium by conversion of cholesterol to coprostanol *via* indirect cholesterol reduction pathway (Freier et al., 1994; Juste and Gérard, 2021). Comparative species-level compositional change of *Bifidobacterium* and *Eubacterium* showed that while total *Bifidobacterium* including *B. animalis* and *B. longum* maintained high level in Week 5 and Week 9 (Supplementary Figure S2A), *E. coprostanoligenes* was detected high level in Week 1 but diminished in Week 5 and Week 9 (Supplementary Figure S2B), suggesting that it was relatively inhibited or not detected even in HFD condition when *Bifidobacterium* was supplemented (Supplementary Figure S2B). In addition, previous supplementation of *Bifidobacterium* reduced total cholesterol by absorption and accumulation of cholesterol in the bifidobacterial cells (Figures 3B, 4A), supporting the cholesterol reduction by *Bifidobacteirium*, not *E. coprostanoligenes* in this condition.

Recent microbiome analysis of human feeding study with konjaku revealed that *Lactococcus* and *Eubacterium brachy* group were significantly reduced, suggesting that they may be positively correlated with obesity (Li et al., 2022). Comparative species-level compositional change of *Lactococcus* and *E. brachy* showed that they were inhibited and reduced even in HFD condition, probably due to the supplementation of *Bifidobacterium* (Supplementary Figures S2B,C), suggesting the probable anti-obesity activity of *B. longum* BCBL-583. However, this activity still remains to be evaluated experimentally in the near future.

### Correlation Analysis of the Microbiome and Biomarkers

**Figure 7:**
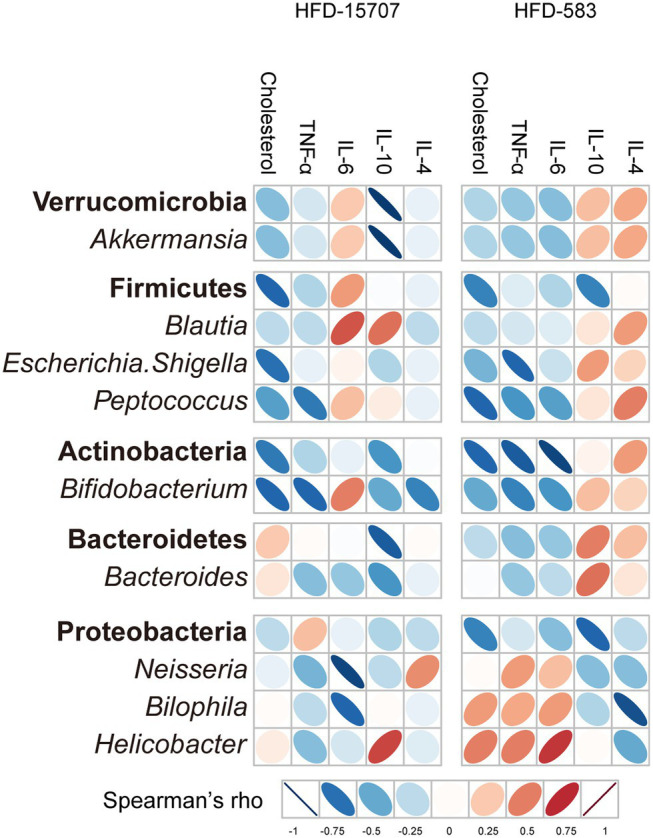
Spearman’s correlation analysis between selected phyla/genera and cholesterol/immune response results of ATCC 15707 and BCBL-583 in HFD mouse models after 9 weeks. Cholesterol and inflammatory cytokines are correlated with selected phyla and genera bacteria.

The Spearman’s correlation analysis between the microbial composition and inflammatory cytokine levels/total-cholesterol was performed using the data from the samples collected at Week 9. The correlation patterns between the HFD-15707 and HFD-583 groups were quite different (Figure 7). In both the HFD-15707 and HFD-583 groups, the correlations of Verrucomicrobia, Firmicutes, and Actinobacteria are inversely proportional to the total cholesterol, indicating that these phyla and the associated genera may be involved in the total cholesterol reduction. However, the correlations of *Bacteroides*, *Neisseria*, *Bilophila*, and *Helicobacter* may be proportional to the total cholesterol. Especially, the correlation of *Bacteroides* with cholesterol in the HFD-15707 group is slightly higher than that in the HFD-583 group, probably explaining why the total cholesterol reduction activity of HFD-583 is slightly higher than that of HFD-15707 (Figure 4). Furthermore, three interesting genera (*Neisseria*, *Bilophila*, and *Helicobacter*) have relatively proportional correlations with total cholesterol in both HFD groups, but they may not increase the total cholesterol level probably because the administration of *B. longum* inhibits their growth like in the previous compositional change analyses (Figure 6F).

While the correlations between pro-inflammatory cytokines and anti-inflammatory cytokines are relatively clear in the HFD-583 group, they are not clearly understood in the HFD-15707 group. In the HFD-583 group, the phyla of Verrucomicrobia, Firmicutes, Actinobacteria, and even Bacteroidetes have inversely proportional correlations with pro-inflammatory cytokines, but proportional correlations with anti-inflammatory cytokines, suggesting that they may have anti-inflammation activities in the HFD-583 group (Figure 7). However, Proteobacteria and the associated genera have inverse correlations with those cytokines in the HFD-583 group. Because the previous compositional change analysis showed that *B. longum* BCBL-583 feeding showed a significant growth inhibition, this inverse correlation may support the anti-inflammation activity in the HFD-583 group. In the HFD-15707 group, these correlations are unclear. However, all selected bacteria in Verrucomicrobia, Firmicutes, and Actinobacteria in the HFD-15707 group have proportional correlations with the pro-inflammatory cytokine IL-6, while those bacteria have inversely proportional correlations in the HFD-583 group, suggesting that *B. longum* BCBL-583 may have a higher anti-inflammation activity than that of *B. longum* ATCC 15707 (Figure 7). The cytokine assay for IL-6 between the HFD-15707 and HFD-583 groups supports this result (Figure 5A). Therefore, these analysis results suggest that the microbial composition and inflammatory cytokine/total-cholesterol due to the administration of *B. longum* in the HFD mice group may be closely related.

## Discussion

To develop a new probiotic strain, *B. longum* BCBL-583 was initially isolated and its functional characteristics of the strain BCBL-583 were evaluated *in vitro*, showing tolerance activities under various oxygen and heat conditions and general probiotic properties including mucin adhesion, gastric/bile acid tolerance, and cholesterol reduction compared to the *B. longum* type strain, ATCC 15707 (Yi et al., 2018). Based on these probiotic effects, strain BCBL-583 may be a good candidate as a new probiotic strain for functional food applications. However, it is necessary to understand these probiotic characteristics and to confirm its safety in humans on molecular and genomic levels.

A previous *in vitro* oxygen tolerance test showed that the strain BCBL-583 has a limited oxygen tolerance activity even at 60 or 120 rpm shaking incubation, even though bifidobacteria are obligate anaerobic bacteria (Yi et al., 2018). In general bacteria, this oxygen tolerance may be due to specific enzyme activities to reduce reactive oxygen species (ROS) including NADH oxidase, NADH peroxidase, and superoxide dismutase (SOD; Lee and O’Sullivan, 2010). However, while bifidobacteria have NADH oxidase to convert ROS to H_2_O_2_, they have no NADH peroxidase or SOD to reduce H_2_O_2_ to H_2_O (Tanaka et al., 2018). To compensate for the reduction of H_2_O_2_, it was reported that bifidobacteria have an alternative reduction mechanism using the thioredoxin system (Arnér and Holmgren, 2000). Bifidobacteria have insufficient NADH production in an anaerobic environment, and this alternative hydrogen peroxide reduction system without NADH peroxidase or SOD may be responsible for this limited oxygen tolerance activity (Lee and O’Sullivan, 2010).

Bifidobacteria are one of the promising probiotic bacteria, widely used as various functional food ingredients. However, food processing conditions like heat treatment are too tough for them to survive in the food products. Therefore, a temperature tolerance activity may be required for the application of bifidobacteria in food products. Previously, *B. longum* BCBL-583 showed a survival rate of 78.61% at 45°C in a heat tolerance test (Yi et al., 2018). This heat tolerance activity may be due to the heat shock tolerance mechanism in bifidobacteria. The genome of strain BCBL-583 carries temperature stress-related gene analogs such as chaperone family genes (Hsp100, Hsp70, and Hsp60). In the expression of the chaperon family, the HrcA-CIRCE (controlling the inverted repeat of chaperon expression) system is involved in the regulation of Hsp70 and Hsp60 chaperon family genes. In the genome of *B. longum* BCBL-583, CIRCE motifs were also found upstream of the *hrcA* gene and *groES* with the consensus repeat sequence of TTAGCACTC-N_9_-GAGTGCTAA (Baldini et al., 1998). The CIRCE-like motif was also found in the promotor region upstream of *groEL* with one mismatch sequence of TTAGCACTC-N_9_-GAGTGCCAA. Therefore, temperature stress induces the expression of *hrcA*, *groES*, and *groEL*, and these gene expressions are regulated by HrcA. In addition, transcriptional regulator gene (*clgR*) was found for regulation of the Hsp100 chaperon family genes (Clp gene cluster). The consensus motif sequence of CGCT-N_4_-GCCNA was identified upstream of the *clpP1* gene to regulate expression (Ventura et al., 2005). Therefore, these chaperone family genes may be actively expressed in heat stress conditions.

Since the first bacteriocin-like compound bifidin from *B. bifidum* NCDC 1452 was detected in 1984 (Anand et al., 1984), several bacteriocin-like compounds from bifidobacteria have been found and analyzed (Martinez et al., 2013). However, only two compounds, bifidocin B and bisin, have been purified and well characterized on a molecular level (Yildirim and Johnson, 1998; Lee et al., 2011). These compounds were identified as antimicrobial peptides by proteolysis assay, and their amino acid sequences were determined. In addition, they could be induced by auto-induction, which is one of the representative characteristics of bacteriocin. The complete genome sequence of *B. longum* DJO10A (CP000605) revealed a bacteriocin gene cluster consisting of three parts: a two-component regulatory system (*lanR2K*), a bisin-encoding gene (*lanA*), and a bisin processing gene cluster (*lanR1DMIT*; Lee et al., 2008, 2011). This two-component system recognizes a specific signal compound like host-producing bacteriocin and phosphorylates the *lanR1* transcription regulator for activation of gene expression in the bisin-encoding gene and the bisin processing gene cluster. After production of the bisin prepeptide, the LanD and LanM bisin prepeptide modification enzymes form the specific structure of the bisin prepeptide. The leader peptide of this modified bisin prepeptide is removed and transferred to the outer membrane for activation and secretion of bisin by the LanT bisin transporter with a protease domain. In addition, the LanI immunity protein endows a bisin resistance activity to the host strain (Lee et al., 2011). However, bisin is not produced in a broth medium but only on an agar medium condition. Interestingly, the regulatory genes (*lanR2KR1*) are constituently expressed, but the bisin-encoding gene and bisin processing gene cluster are expressed only on an agar medium condition (Lee et al., 2011). This specific gene expression pattern may be involved in the specific property of bifidobacteria regarding adhesion on the mucosal layer in the human gut. Further comparative genome analysis of the intestinal bifidobacteria showed a homologous bacteriocin gene cluster in their genomes but no gene cluster in the commercial bifidobacterial genomes, suggesting that the bacteriocin gene cluster may be removed in the commercial bifidobacteria, and it can be one of the distinct characteristics of intestinal bifidobacteria (Lee et al., 2008; Lee and O’Sullivan, 2010). Notably, the genome of the human gut-originated strain BCBL-583 also has a homologous bacteriocin gene cluster, and this strain showed an anti-*H. pylori* activity, suggesting that this strain may have a similar bacteriocin production ability for competitive survival against the gut microbiota. Furthermore, this bacteriocin gene cluster shares a high DNA sequence similarity to those of other human gut-originated *B. longum* subsp. *longum* strains including JSRL02 (CP046514, 99.9%), Jih1 (AP022868, 99.88%), and HN001 (CP069278, 99.62%), supporting this result.

To validate these strain-specific characteristics *in vivo*, administration of bifidobacteria to a specific mouse model and its subsequent functional properties need to be verified. For this study, two major research objectives have to be accomplished: (1) cholesterol reduction *in vivo* by bifidobacteria and (2) immune regulation from obesity *in vivo* by bifidobacteria. To test these two major functionalities of bifidobacteria, a specific mouse model needs to be developed. In this study, a mouse model was developed using an HFD containing low carbohydrates, high lipids and high cholesterol (Supplementary Table S2). Continuous feeding of the HFD to the mice (HFD mouse model) for 8 weeks resulted in obese mice which gained more weight (22.8%) than that of the ND fed group (ND mouse model). This HFD mouse model has about 60% more blood cholesterol and higher pro-inflammatory cytokines (about 10-fold higher TNF-α and 3-fold higher IL-6) compared to the ND mouse model (Figures 5, 6), indicating that the HFD mouse model is suitable for *in vivo* evaluation of cholesterol reduction and immune regulation from obesity by bifidobacteria in this study.

To investigate the cholesterol reduction effect of *B. longum* in the mouse model, HFD feed with 0.023% cholesterol (Supplementary Table S2) was administered together with *B. longum* BCBL-583. Once absorbed from the intestine, cholesterol is transferred to the liver in the form of chylomicrons with lipoprotein. And then, the liver secrets triglyceride-rich very low-density lipoproteins (VLDL) for the transportation of triglycerides to peripheral tissues. After hydrolysis of the triglycerides by lipoprotein lipase in the muscle and adipose tissues, the VLDL particles are reduced to low-density lipoprotein (LDL) particles. In the cells of the peripheral tissue, cholesterol uptake from LDL is used as a cell membrane component and as solubilizers of lipid. The excess cholesterol is transferred to the liver in the form of high-density lipoproteins (HDL) for reverse cholesterol transport. In the liver, cholesterol is reused to generate bile acid secreted into the intestine (Kriaa et al., 2019). Because of this coordinate cholesterol transport system, the HFD may increase the absorption of cholesterol from the intestine and break the balance of LDL and HDL (Kwan et al., 2007). To evaluate the cholesterol reduction effect *in vivo* and the recovery of the balance of LDL and HDL by bifidobacteria, the concentrations of total cholesterol, HDL-cholesterol, and LDL-cholesterol in serum were determined after sacrificing the mice. This study showed that BCBL-583 can reduce the extracellular cholesterol through *in vitro* adsorption and accumulation of cholesterol in the cells. In addition, the subsequent *in vivo* HFD mouse model study revealed that this strain reduced the total cholesterol and LDL-cholesterol but did not reduced the HDL-cholesterol in the blood samples, probably due to the adsorption and accumulation of cholesterol in the cells by BCBL-583.

This study showed that HFD increased body weight, total cholesterol, and inflammatory cytokines, indicating that obesity, cholesterol, and inflammation might be correlated. However, feeding of bifidobacteria decreased these all negative effects in HFD mouse model. Previous feeding study of HFD mouse model with *B. breve* B-3 showed reductions of body weight and total cholesterol, and even increment of bifidobacteria in the gut, suggesting cholesterol lowering effect by bifidobacteria probably due to their negative correlation between bifidobacteria and total cholesterol like this study (Kondo et al., 2014; Figure 7). In addition, quantitative analysis of specific gut bacteria between normal and obese human subjects using qPCR revealed reduction of bifidobacteria in obese people, suggesting anti-obesity effect by bifidobacteria (Million et al., 2011). Furthermore, another feeding study of HFD mouse model with *Bifidobacterium pseudocatenulatum* CECT 7765 also showed anti-inflammation activity of *B. pseudocatenulatum* CECT 7765 by reduction of pro-inflammatory cytokines but increment of anti-inflammation cytokines, supporting this study (Cano et al., 2013; Figure 5). Therefore, these studies support potential anti-obesity, cholesterol reduction, and anti-inflammation effects by bifidobacteria.

To further understand the effect of *Bifidobacterium* administration in the HFD mouse model, metagenomics analysis of the fecal samples after ingestion of *Bifidobacterium* was conducted. As discussed previously, HFD feed contains low carbohydrates and high lipids (Supplementary Table S2). Therefore, the HFD feed changed the composition of the gut microbiota in the HFD mouse model, such as the decrease of *Akkermansia* and increase of *Blautia* in Week 5 and Week 9 (Figure 6). However, *Bifidobacterium* increased in those weeks unlike what was expected, due to continuous feeding with the HFD feed. This increased *Bifidobacterium* inhibited the growth of *Helicobacter* during these feeding periods. Previously, it was reported that human trials of *B. animalis* subsp. *lactis* BB-12 reduced the density of *H. pylori* in the gastric environment of humans, supporting this result (Wang et al., 2004). However, while administration of *Bifidobacterium* may not have an impact on the modulation of the overall gut microbiota, the β-diversity analyses revealed that ATCC 15707 and BCBL-583 may have an important role in the modulation of the gut microbiota composition in the HFD mouse model (Figure 6). Based on these microbiome results, among these bifidobacteria, *B. longum* BCBL-583 may be a key strain to modify the composition of the gut microbiota in the HFD mouse to a health-promoting composition even in the HFD condition. In addition, although the role of *Blautia* in gut microbiota is not clearly understood yet, the recent microbiome studies often revealed the compositional change of *Blautia*, suggesting its possible correlation with obesity by HFD feeding. Recently, it was found that lean children have high composition of *Blautia* and suggested that the depletion of *B. luti* and *B. wexlerae* in obese children may be associated with intestinal inflammation by determination of inflammatory biomarkers (Benítez-Páez et al., 2020). However, other recently published papers (Duan et al., 2021; Jo et al., 2021) and this study revealed that HFD feeding to mouse or human increased the genus *Blautia*, suggesting that obesity by HFD feeding changed the composition of gut microbiota including *Blautia* and even metabolomic profiles. Based on these conflicting results, it is still necessary to elucidate the characteristics of *Blautia* (or even specific species) and to perform the direct feeding of this bacterium to obese/lean mouse model or human participants to clarify the role of this bacterium for correlation with human health in the near future.

In addition, compositional change analysis of gut microbiota in HFD condition by supplementation of *B. longum* BCBL-583 was performed in genus as well as species level. It has been known that *E. coprostanoligenes* has cholesterol reduction activity by bioconversion of cholesterol to coprostanol *via* indirect cholesterol reduction pathway. This pathway consists of oxidation (to 5-cholesten-3-one), isomerization (to cholestenone), and reduction steps (to coprostanone and then coprostanol as an end product; Juste and Gérard, 2021). This coprostanol is not absorbed by human intestinal system, so it is removed by fecal defecation, indicating the cholesterol reduction (Freier et al., 1994; Juste and Gérard, 2021). However, a recent paper showed that *Bifidobacterium* does not have the bioconversion activity of cholesterol to coprostanol (Cardona et al., 2000; Kenny et al., 2020). This study confirmed that the cholesterol reduction activity of *B. longum* BCBL-583 is due to absorption and accumulation of cholesterol inside its cells (Figure 3B). The compositional change analysis of *Bifidobacterium* and *E. coprostanoligenes* revealed that *E. coprostanoligenes* may be inhibited by *Bifidobacterium*, even though they grow in HFD condition containing high cholesterol amount, suggesting the possible competition of cholesterol acquisition between these two gut bacteria with different cholesterol reduction mechanisms. The majority of *Bifidobacterium* comparing to very low composition of *E. coprostanoligenes* in the gut microbiota probably suggests that most of cholesterol may be reduced by *Bifidobacterium*, not by *E. coprostanoligenes.* In addition, it was previously reported that *E. coprostanoligenes* does not grow under absence of cholesterol and lecithin (Freier et al., 1994). Therefore, supplementation of *Bifidobacterium* mainly reduced cholesterol and probably inhibited *E. coprostanoligenes* in the gut environment *via* this competition of cholesterol reduction even in HFD condition. As previously discussed in the Results, it was suggested that *Lactococcus* may be positively correlated with HFD or obesity (Li et al., 2022). The comparative species-level compositional change analysis of *Lactococcus lactis* and *Lactococcus* unidentified species revealed that they significantly increased in the HFD condition, supporting this (Supplementary Figure S2C). Previous mouse feeding study with HFD also showed that *Lactococcus* significantly increased in HFD up to 36 weeks, comparing to low-fat diet (LFD), consistent with the result in this study (Zeng et al., 2016). However, *Lactococcus lactis* and *Lactococcus* unidentified species were reduced and inhibited in HFD condition when each *B. longum* strain was supplemented (ATCC 15707 or BCBL-583; Supplementary Figure S2C). Similar result was previously reported, revealing that *Lactococcus* increased in HFD condition but it was significantly reduced even in HFD condition by the supplementation of *Bifidobacterium*, substantiating our result (Moya-Pérez et al., 2015). Subsequent correlation analysis between *Lactococcus* and *Bifidobacterium* in HFD condition showed that they are negatively correlated. Therefore, while *Lactococcus* may prefer HFD condition, the supplementation of *Bifidobacterium* may reduce or inhibit *Lactococcus*, probably associated with obesity, even in HFD condition, suggesting the possible anti-obesity activity of *Bifidobacterium*.

**Figure 8:**
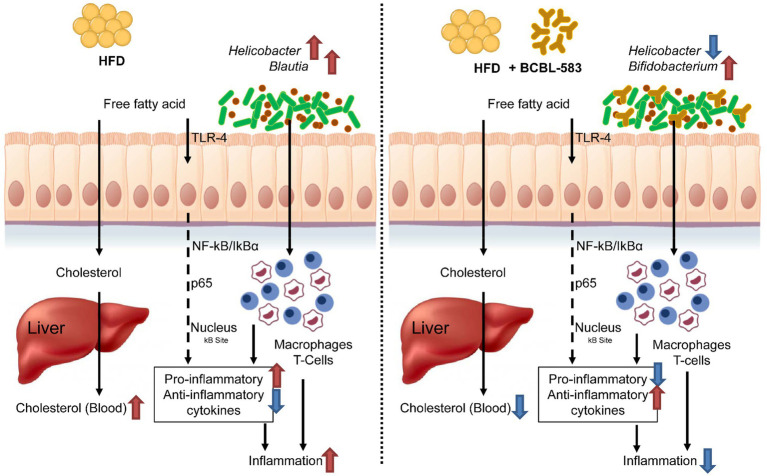
Summary on the probiotic effect of *B. longum* BCBL-583 in HFD mouse model.

As a summary of the overall results, administering HFD causes increased blood cholesterol, inflammation, and potential pathogens in the mouse gut microbiota of the HFD only group, indicating an undesirable direction for health promotion. However, administration of the strain BCBL-583 in the HFD feeding group (HFD-583) solved this undesirable problem regarding obesity caused by the administered HFD and resulted in a reduction of blood cholesterol, inflammation and an increase of *Bifidobacterium* in the mouse gut microbiota (Figure 8). Therefore, based on this study, BCBL-583 may be a good probiotic candidate strain for health promotion *via* various functional food applications.

## Data Availability Statement

The datasets presented in this study can be found in online repositories. The names of the repository/repositories and accession number(s) can be found in the article/Supplementary Material.

## Author Contributions

Y-TK, C-HK, Y-SS, and J-HL: conceptualization. Y-TK, C-HK, HK, and J-HL: methodology. Y-TK, C-HK, J-GK, and JC: validation, formal analysis, and investigation. Y-TK, C-HK, and J-GK: data curation and visualization. Y-SS: resources. Y-TK, C-HK, J-GK, HK, and J-HL: writing—original draft. Y-TK, J-GK, HK, and J-HL: writing—review and editing. Y-SS, HK, and J-HL: supervision. J-HL: project administration and funding acquisition. All authors contributed to the article and approved the submitted version.

## Funding

This research was supported by the Food Research Center, Binggrae, Co., Ltd., and the Korea Institute of Planning and Evaluation for Technology in Food, Agriculture, and Forestry (IPET) through the Agricultural Microbiome R&D Program (no. 918022-04-HD020). And, it was also supported by the Cooperative Research Program for Agriculture Science and Technology Development (project no. PJ0158652021) and Rural Development Administration, South Korea.

## Conflict of Interest

C-HK and Y-SS were employed by Food Research Center, Binggrae Co., Ltd.

The remaining authors declare that the research was conducted in the absence of any commercial or financial relationships that could be construed as a potential conflict of interest.

## Publisher’s Note

All claims expressed in this article are solely those of the authors and do not necessarily represent those of their affiliated organizations, or those of the publisher, the editors and the reviewers. Any product that may be evaluated in this article, or claim that may be made by its manufacturer, is not guaranteed or endorsed by the publisher.
