# Supplementary material for: In vivo Trial of Bifidobacterium longum Revealed the Complex Network Correlations Between Gut Microbiota and Health Promotional Effects
Source: Front Microbiol. 2022 Jun 17;13:886934. doi: 10.3389/fmicb.2022.886934 (PMC9247516; doi:10.3389/fmicb.2022.886934)
Supplement: Supplementary file 1 [file Data_Sheet_1.docx]

*In vivo* trial of *Bifidobacterium longum* revealed the complex network correlations between gut microbiota and health promotional effects

**You-Tae Kim^1,2,3,4,†‡^, Chul-Hong Kim^5,6,‡^, Joon-Gi Kwon^1,2,3,4^, Jae Hyoung Cho^7^, Young-Sup Shin^6^, Hyeun Bum Kim^7*^, and Ju-Hoon Lee^1,2,3,4*^**

^1^ Department of Food and Animal Biotechnology, Seoul National University, Seoul, South Korea

^2^ Department of Agricultural Biotechnology, Seoul National University, Seoul, South Korea

^3^ Center for Food and Bioconvergence, Seoul National University, Seoul, South Korea

^4^ Research Institute of Agriculture and Life Science, Seoul National University, Seoul, South Korea

^5^ Department of Food Science and Biotechnology, Graduate School of Biotechnology, Kyung Hee University, Yongin, South Korea

^6^ Food Research Center, Binggrae Co., Ltd, Namyangju, South Korea

^7^ Department of Animal Resources Science, Dankook University, Cheonan, South Korea

**^†^** Present address: One Shields Ave., Department of Food Science & Technology, University of California, Davis, CA 95616, USA

^‡^ These authors have contributed equally to this work and share first authorship

*** Correspondence:**Dr. Ju-Hoon Lee / juhlee@snu.ac.kr

Dr. Hyeun Bum Kim / hbkim@dankook.ac.kr

Keywords: *Bifidobacterium longum*, Cholesterol reduction, Anti-inflammation, Gut microbiota, Obesity.**Table S1. Primer sets used in qRT-PCR**

| **Name** | **Type** | **Target genes** | **Sequence (5’ to 3’)** | **Amplicon size (bp)** |
| --- | --- | --- | --- | --- |
| RecA_F | Forward primer | *recA*  (BCBL_01217) | CTCCGTGCGTCTCGACATCC | 173 |
| RecA_R | Reverse primer |  | TGTCGATCACCGAGCCCTCA |  |
| bsh_F | Forward primer | *bsh*  (BCBL_01011) | AATGTGTCGCGCCTGTTCCA | 170 |
| bsh_R | Reverse primer |  | ATGGCGTAGGAACGGATGGC |  |

**Table S2. The formula composition of ND and HFD fed to mice**

| Ingredient | Normal diet (D12450B^a^) | | High fat diet (D12492^a^) | |
| --- | --- | --- | --- | --- |
|  | g | kcal% | g | kcal% |
| Casein, 30 mesh | 200 | 800 | 200 | 800 |
| L-Cysteine | 3 | 12 | 3 | 12 |
| Corn starch | 315 | 1260 | 0.00 | 0 |
| Maltodextrin 10 | 35 | 140 | 125 | 500 |
| Sucrose | 315 | 1400 | 68.8 | 275.2 |
| Cellulose, BW200 | 50 | 0 | 50 | 0 |
| Soybean oil | 25 | 225 | 25 | 225 |
| Lard^b^ | 20 | 180 | 245 | 2205 |
| Mineral mix S10026 | 10 | 0 | 10 | 0 |
| Dicalcium phosphate | 13 | 0 | 13 | 0 |
| Calcium carbonate | 5.5 | 0 | 5.5 | 0 |
| Potassium citrate, 1H_2_O | 16.5 | 0 | 16.5 | 0 |
| Vitamin mix V10001 | 10 | 40 | 10 | 40 |
| Choline bitartrate | 2 | 0 | 2 | 0 |
| FD%C yellow dye #5 | 0.05 | 0 | 0.05 | 0 |
| Total | 1055.05 | 4057 | 773.85 | 4057 |

^a^, The information of formula and calculated kcal% was obtained from Research Diet.

^b^, Typical analysis of cholesterol in lard = 72 mg per 100 grams. D12450B contains 14.4 mg/4057 kcal (0.0014%; wt/wt) and D12492 contains 176.4 mg/4057 kcal (0.0228%; wt/wt).

**Table S3.** Probiotic effect related ORFs and clusters

| **Functional categories and gene annotation** | | **Locus tag** | |
| --- | --- | --- | --- |
| **Carbohydrate degrading enzyme** | | | |
|  | 4-α-glucanotransferase (amylomaltase) | BCBL_00104, BCBL_01752 | |
|  | α-galactosidase | BCBL_01962 | |
|  | β-galactosidase | BCBL_00374, BCBL_00485,  BCBL_01905 | |
|  | α-L-arabinofuranosidase | BCBL_00487, BCBL_00522,  BCBL_01705 | |
|  | α-mannosidase | BCBL_01302 to BCBL_01304 | |
|  | 1,4-β-xylanase | BCBL_01787 | |
|  | α-xylosidase | BCBL_01419 | |
|  | GH family 3 (β-glucosidase) | BCBL_01533, BCBL_01556,  BCBL_00636 | |
|  | GH family 5 (Cellulase) | BCBL_01540 | |
|  | GH family 13 (α-glucosidase) | BCBL_00102 | |
|  | GH family 31 (α-glucosidase) | BCBL_00109 | |
|  | GH family 32 (β-fuctofuranosidase) | BCBL_00440, BCBL_01371 | |
|  | GH family 43 | BCBL_01788 | |
|  | GH family 53 | BCBL_00376 | |
|  | Glycogen debranching enzyme | BCBL_01749 | |
|  | Glycogen phosphorylase | BCBL_00044 | |
| **Adhesion (anchoring and pilus construction)** | | | |
|  | Signal peptidase I and II | BCBL_00356, BCBL_01033  BCBL_01353 | |
|  | Signal recognition particle (SRP) pathway | BCBL_00185, BCBL_00322 | |
|  | Protein translocase subunit | BCBL_01133, BCBL_01213  BCBL_01717, BCBL_01824 | |
|  | Sortase A | BCBL_00049, BCBL_00132,  BCBL_00750, BCBL_01976 | |
|  | Membrane protein with signal peptide and LPXTG motif | BCBL_00237, BCBL_00751,  BCBL_00752, BCBL_01804,  BCBL_01805, BCBL_01936,  BCBL_01977 | |
|  | Tight adhesion (*tad*) locus | BCBL_00122 to BCBL_00128 | |
|  | Prepilin peptidase (*tadV*) | BCBL_00921 | |
|  | Prepilin (*fimA*) | BCBL_01977 | |
| **Bacteriocin** | | | |
|  | Bacteriocin cluster | BCBL_01793 to BCBL_01800 | |
|  | Bacteriocin-like peptide | BCBL_01798 | |
| **Oxygen tolerance** | | | |
|  | NADH oxidase | BCBL_01944 | |
|  | Thioredoxin systems | BCBL_00026, BCBL_00418, BCBL_02004 | |
| **Heat tolerance** | | |  |
|  | HrcA-CIRCE | BCLB_01124 |  |
|  | Heat-shock proteins (*groESEL*) | BCBL_01771, BCBL_01482 |  |
|  | Clp transcriptional regulator | BCBL_01219 |  |
|  | Clp gene cluster | BCBL_00843 to BCBL_00845 |  |
| **Cholesterol reduction** | | | |
|  | Bile salt hydrolase | BCBL_01011 | |
|  | Aldo/keto reductase | BCBL_01340 | |
|  | ABC transporter | BCBL_01435, BCBL_01923,  BCBL_00495 | |
|  | Symporter | BCBL_00099 | |
|  | PTS system | BCBL_01686 | |

**Table S4.** Identification and compositional change of gut microbiota in the species level

| Genus | Species | Control | | | HFD | | | HFD-707 | | | HFD-583 | | | |
| --- | --- | --- | --- | --- | --- | --- | --- | --- | --- | --- | --- | --- | --- | --- |
|  |  | Week 1 | Week 5 | Week 9 | Week 1 | Week 5 | Week 9 | Week 1 | Week 5 | Week 9 | Week 1 | Week 5 | Week 9 |  |
| *Acinetobacter* | *baumannii* | 0 | 0 | 0 | 0 | 0 | 2 | 0 | 0 | 0 | 0 | 0 | 0 |  |
|  | *calcoaceticus* | 5 | 0 | 0 | 0 | 0 | 0 | 0 | 0 | 0 | 0 | 0 | 0 |  |
|  | Unidentified species | 0 | 0 | 0 | 0 | 3 | 2 | 0 | 0 | 0 | 0 | 2 | 2 |  |
| *Anaerobacillus* | NB2006 | 2 | 2 | 0 | 2 | 0 | 0 | 0 | 0 | 0 | 2 | 0 | 0 |  |
| *Bacillus* | *subtilis* | 0 | 0 | 2 | 0 | 0 | 3 | 0 | 0 | 0 | 0 | 2 | 0 |  |
| *Bacteroides* | *ovatus* | 0 | 0 | 0 | 2 | 0 | 0 | 0 | 0 | 0 | 2 | 0 | 0 |  |
| *Bifidobacterium* | *animalis* | 0 | 0 | 0 | 2 | 0 | 37 | 0 | 2 | 3 | 0 | 83 | 52 |  |
|  | *longum* | 2 | 0 | 7 | 0 | 0 | 28 | 0 | 2 | 8 | 0 | 17 | 3 |  |
|  | Unidentified species | 202 | 765 | 11685 | 38 | 363 | 13740 | 342 | 1050 | 7593 | 233 | 23782 | 10278 |  |
| *Citrobacter* | *freundii* | 0 | 0 | 0 | 0 | 0 | 3 | 0 | 0 | 0 | 0 | 0 | 0 |  |
| *Enteroccoccus* | *faecalis* | 3 | 3 | 0 | 0 | 0 | 0 | 0 | 2 | 12 | 0 | 0 | 0 |  |
| *Escherichia* | *coli* | 0 | 0 | 0 | 0 | 0 | 2 | 0 | 0 | 0 | 2 | 0 | 0 |  |
|  | Unidentified species | 2 | 2 | 2 | 0 | 2 | 8 | 2 | 0 | 23 | 2 | 3 | 2 |  |
| *Eubacterium* | *brachy* | 5 | 13 | 20 | 0 | 18 | 37 | 0 | 8 | 22 | 2 | 12 | 18 |  |
|  | *coprostanoligenes* | 267 | 32 | 5 | 23 | 0 | 163 | 33 | 10 | 80 | 243 | 0 | 0 |  |
|  | *hallii* | 2 | 0 | 0 | 2 | 2 | 0 | 0 | 2 | 0 | 0 | 0 | 2 |  |
|  | *nodatum* | 48 | 18 | 12 | 20 | 22 | 200 | 3 | 5 | 132 | 7 | 17 | 162 |  |
|  | *oxidoreducens* | 72 | 45 | 2 | 127 | 202 | 40 | 55 | 697 | 105 | 67 | 58 | 87 |  |
|  | *ruminantium* | 0 | 0 | 0 | 3 | 0 | 0 | 0 | 0 | 0 | 0 | 0 | 0 |  |
|  | *ventriosum* | 2 | 2 | 0 | 10 | 33 | 3 | 2 | 13 | 2 | 5 | 8 | 0 |  |
|  | *xylanophilum* | 10 | 5 | 18 | 28 | 108 | 62 | 160 | 155 | 18 | 37 | 135 | 0 |  |
| *Klebsiella* | *pneumoniae* | 0 | 0 | 0 | 0 | 2 | 0 | 0 | 0 | 0 | 0 | 0 | 0 |  |
| *Lactobacillus* | *gasseri* | 0 | 0 | 3 | 0 | 0 | 0 | 0 | 0 | 0 | 2 | 0 | 0 |  |
|  | *reuteri* | 0 | 0 | 0 | 0 | 0 | 0 | 0 | 0 | 0 | 0 | 0 | 2 |  |
|  | Unidentified species | 10 | 18 | 37 | 8 | 12 | 33 | 0 | 0 | 7 | 3 | 10 | 8 |  |
| *Lactococcus* | *lactis* | 3 | 8 | 2 | 0 | 5 | 30 | 0 | 5 | 7 | 0 | 12 | 8 |  |
|  | Unidentified species | 378 | 1188 | 588 | 150 | 995 | 2662 | 35 | 253 | 820 | 82 | 798 | 705 |  |
| *Micrococcus* | *luteus* | 0 | 0 | 0 | 0 | 2 | 0 | 0 | 0 | 0 | 0 | 0 | 0 |  |
| *Paenarthrobacter* | *nicotinovorans* | 0 | 0 | 0 | 0 | 2 | 0 | 0 | 0 | 0 | 0 | 0 | 0 |  |
| *Pediococcus* | *acidilactici* | 0 | 0 | 0 | 0 | 0 | 0 | 0 | 0 | 0 | 0 | 0 | 2 |  |
| *Pseudomonas* | *putida* | 0 | 0 | 1 | 0 | 0 | 0 | 0 | 0 | 0 | 0 | 0 | 1 |  |
|  | *stutzeri* | 0 | 0 | 0 | 1 | 0 | 0 | 0 | 0 | 0 | 0 | 3 | 0 |  |
| *Rhodococcus* | *erythropolis* | 0 | 3 | 0 | 0 | 0 | 0 | 0 | 0 | 0 | 0 | 0 | 0 |  |
| *Ruminococcus* | *gauvreauii* | 3 | 0 | 0 | 0 | 3 | 0 | 1 | 18 | 0 | 2 | 0 | 0 |  |
|  | *gnavus* | 0 | 0 | 0 | 0 | 0 | 1 | 2 | 7 | 7 | 0 | 2 | 8 |  |
|  | *torques* | 5 | 0 | 0 | 0 | 1 | 3 | 0 | 17 | 0 | 3 | 2 | 2 |  |
|  | Unidentified species | 5 | 12 | 2 | 15 | 30 | 18 | 13 | 133 | 18 | 18 | 23 | 23 |  |
| *Sporosarcina* | *pasteurii* | 0 | 0 | 1 | 0 | 8 | 18 | 0 | 0 | 3 | 0 | 3 | 0 |  |
| *Staphylococcus* | *aureus* | 0 | 0 | 0 | 0 | 0 | 0 | 0 | 0 | 0 | 0 | 1 | 0 |  |
|  | *epidermidis* | 1 | 0 | 0 | 0 | 0 | 0 | 0 | 0 | 0 | 0 | 0 | 1 |  |
|  | *haemolyticus* | 3 | 0 | 0 | 0 | 2 | 5 | 0 | 0 | 2 | 0 | 0 | 2 |  |
|  | *xylosus* | 2 | 8 | 0 | 0 | 0 | 0 | 0 | 0 | 1 | 0 | 0 | 0 |  |
|  | Unidentified species | 8 | 2 | 2 | 3 | 47 | 48 | 3 | 0 | 10 | 7 | 15 | 22 |  |
| *Stenotrophomonas* | *maltophilia* | 0 | 0 | 0 | 0 | 0 | 0 | 0 | 0 | 0 | 0 | 0 | 1 |  |
| *Streptococcus* | *gallolyticus* | 0 | 0 | 0 | 0 | 0 | 0 | 0 | 0 | 0 | 0 | 0 | 2 |  |
|  | Unidentified species | 13 | 38 | 75 | 3 | 172 | 823 | 0 | 22 | 103 | 8 | 108 | 155 |  |

**
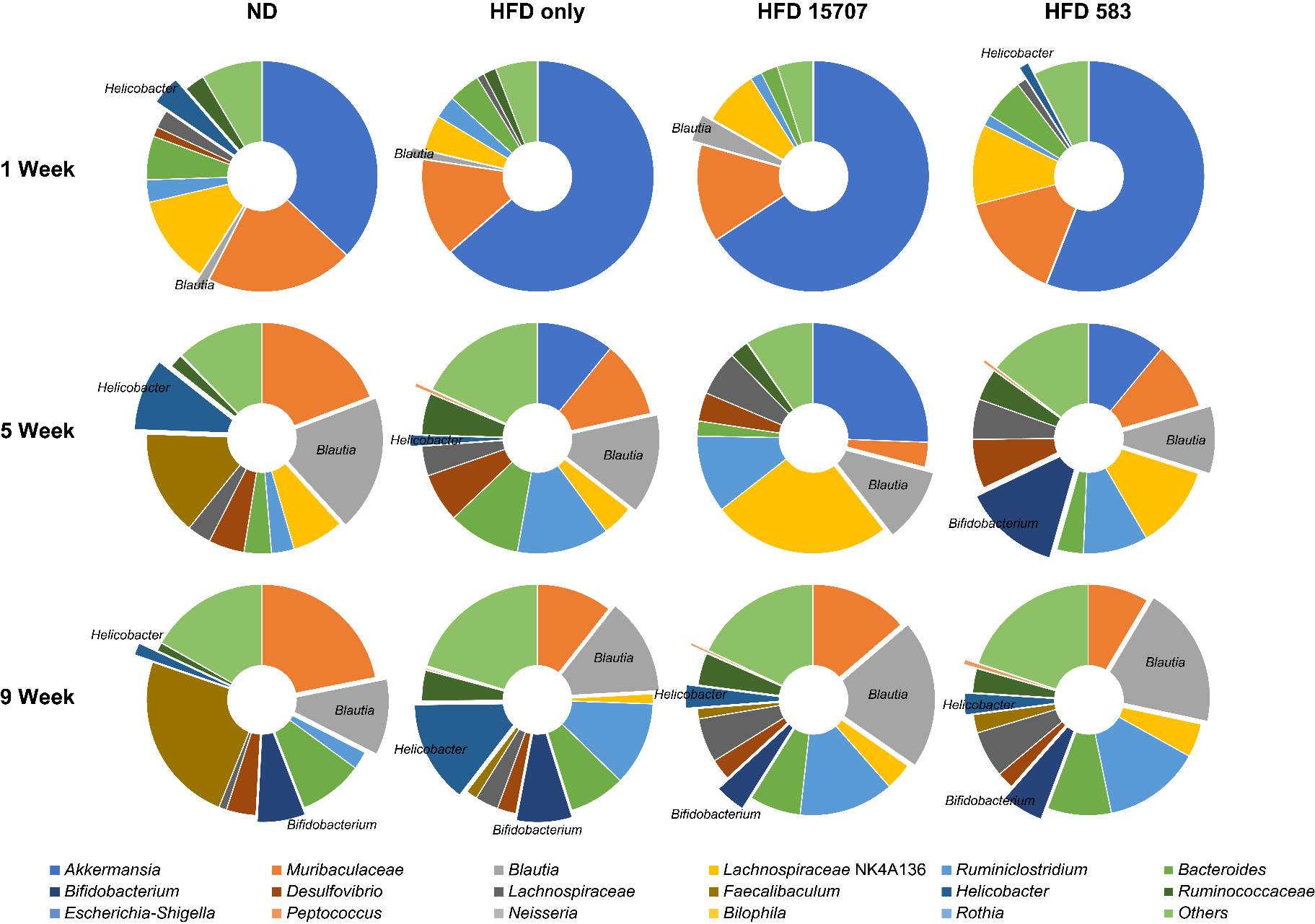
**

**Figure S1.** Compositional analysis of gut microbiota of mouse model in genus level according to week.


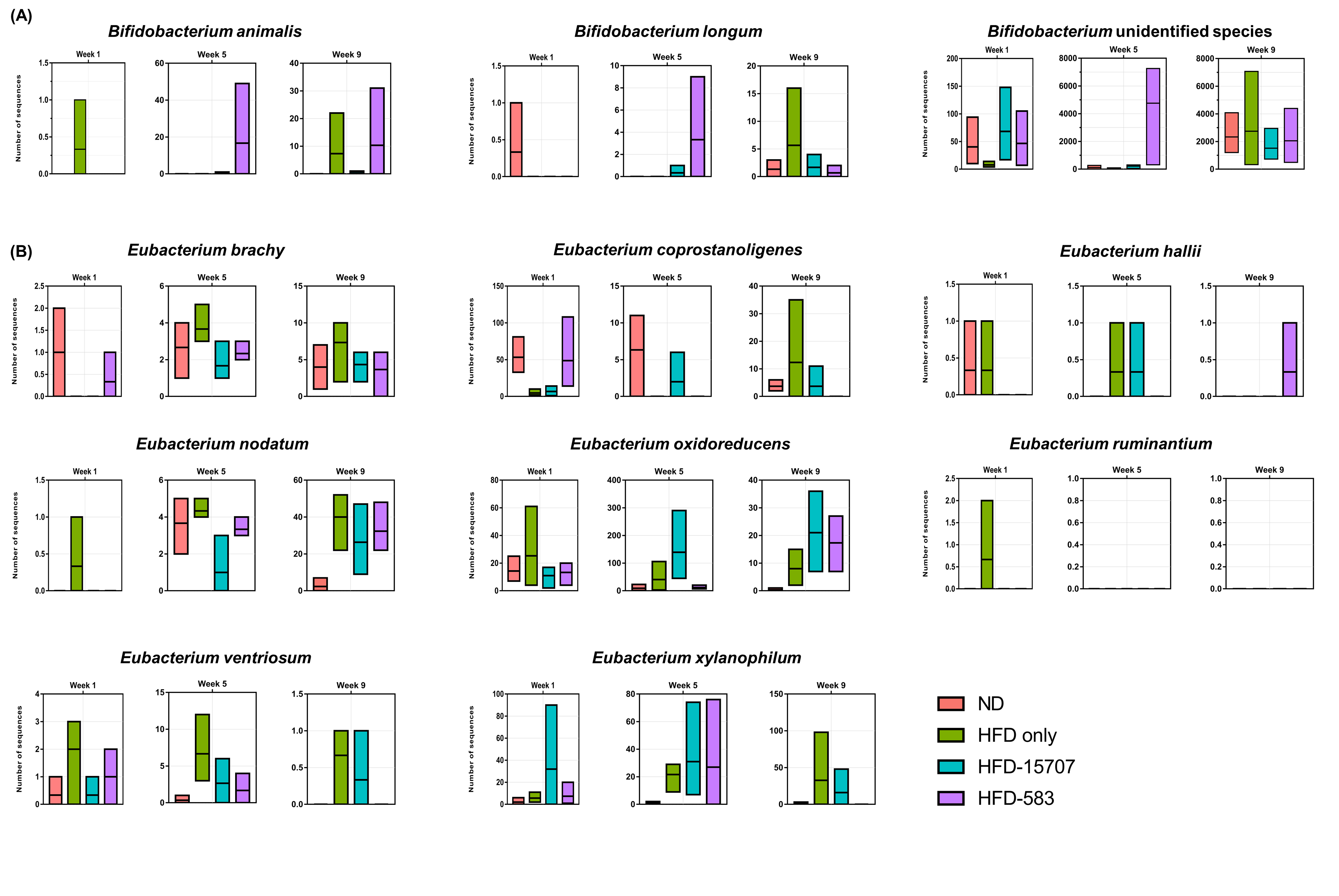


**Figure S2.** Microbial compositional changes of species levels of four groups in Week 1, Week 5, and Week 9


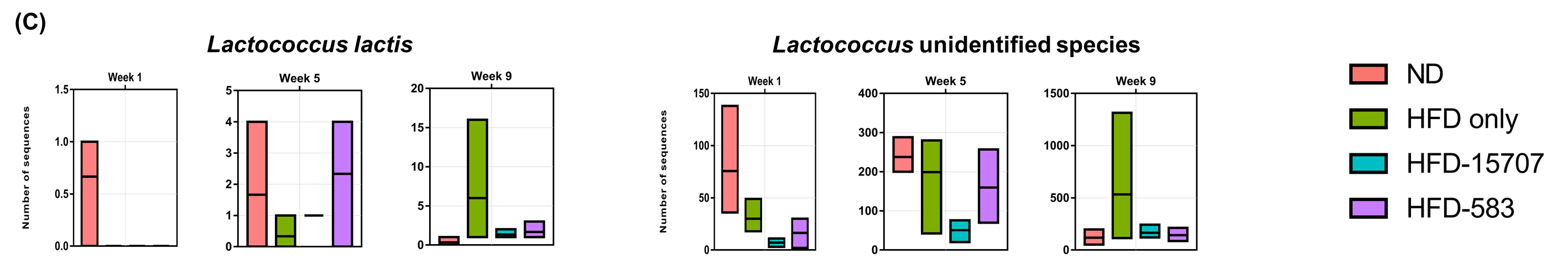


**Figure S2 (cont.)** Microbial compositional changes of species levels of four groups in Week 1, Week 5, and Week 9
